# Associations between dietary behaviours and the mental and physical well-being of Swedish adolescents

**DOI:** 10.1186/s13034-024-00733-z

**Published:** 2024-03-30

**Authors:** Kenisha Russell Jonsson, Cameron Kymani Bailey, Maria Corell, Petra Löfstedt, Nicholas Kofi Adjei

**Affiliations:** 1 School of Public Health and Community Medicine, Institute of Medicine, Gothenburg University, Box 463, Göteborg, 405 30 Sweden; 2 Department of Public Health and Policy, University of Liverpool, Waterhouse Building 2nd Floor, Block F, Liverpool, L69 3GL UK; 3 Leibniz Institute for Prevention Research and Epidemiology - BIPS, Bremen, Germany; 4 Health Sciences Bremen, University of Bremen, Bibliothekstrasse 1, 28359 Bremen, Germany; 5 Jamaica College, 189 Old Hope Road, Kingston, Jamaica

**Keywords:** Eating behaviour, Diet, Mental health, Physical health, Socioeconomic disparities, Adolescents, Obesity, Public health

## Abstract

**Aims:**

This study aims to investigate the association between dietary behaviours, overweight/obesity, and mental health and well-being among Swedish adolescents.

**Methods:**

Data from the 2017/2018 Health Behaviour in School-aged Children (HBSC) survey of 3692 adolescents aged ≈11, ≈13, and ≈15 years was analysed. We evaluated the prevalence and association of dietary behaviours, characterised by daily intake of fruits and vegetables, consumption of sugar-sweetened beverages (SSBs) and sweets once per week, and daily consumption of family meals and breakfast, with socioeconomic and demographic factors. Multivariate logistic regression models (adjusted and unadjusted) were then used to examine the relationship between dietary behaviours, overweight/obesity, and mental health and well-being including psychosomatic complaints, life satisfaction, and school-related pressure.

**Results:**

Boys were more likely to eat breakfast and have meals together with their family than girls, but their fruit and vegetable intake was lower compared to girls. Adolescents with lower socioeconomic status (low family affluence, families perceived ’not well-off’ and two unemployed parents), single-parent households and twoforeign born parents were more likely to engage in poorer dietary behaviours. Daily breakfast, family meals, fruit and vegetable intake were positively associated with mental health and well-being. Specifically, daily family meals were linked to higher life satisfaction, fewer psychosomatic complaints, and reduced school-related pressure. Breakfast emerged as a beneficial dietary habit, associated with higher life satisfaction, and a lower likelihood of psychosomatic complaints, school-related pressure, and overweight/obesity. Fruit and vegetable consumption was consistently associated with better mental health and well-being while associations between SSBs and sweets were mixed.

**Conclusions:**

Improving mental health and well-being, along with tackling the rising rates of mental illness and challenges related to overweight/obesity in adolescents constitute key public health priorities. Implementing policies that promote the intake of fruits and vegetables, reducing the consumption of SSBs and sweets, and emphasizing the value of having breakfast and sharing family meals could offer a cost-effective public health intervention.

**Supplementary Information:**

The online version contains supplementary material available at 10.1186/s13034-024-00733-z.

## Introduction

Adolescence is a pivotal life stage for cultivating healthy dietary behaviours [1, 2] which includes, but is not limited to the quantity, quality, and diversity of foods consumed, but also the regularity of family meals [3, 4] and breakfast consumption [5, 6]. During this phase, individuals begin to make more autonomous food choices, laying the foundation for a wide range of dietary behaviours [7]. The development of healthy dietary behaviours at this stage is cruical given that health behaviours established during adolescence, often persist into adulthood [1, 2]. Sweden’s official nutrition recommendations, based on the World Health Organization (WHO) and Nordic Nutrition Recommendations (NNR), state that adolescents should consume 400 g (four portions) of fruits and vegetables daily [8]. They are advised to limit their consumption of energy-dense and nutrient-poor foods, such as sugar sweetened beverages (SSBs) and sweets, to maintain a healthy diet [8]. In particular, eating more fruits and vegetables may contribute to improvements in nutritional status and health [9]. Additionally, the regularity of breakfast [5, 6, 10] and sharing mealtimes with family [3, 4], are suggested to be indicators of a high intake of micronutrients, and healthy dietary practice in general. Despite these recommedations, studies show that adolescents need to increase their intake of fruits and vegetables [11, 12]. Breakfast consumption has also declined over time and varies by both gender and age [13, 14].

The current dietary behaviours among Swedish adolescents are important and may have wide ranging public health implications. This is because healthy dietary behaviours are integral in the promotion, prevention and treatment of current and future physical and mental health outcomes [15–20]. In particular, engaging in unhealthy dietary behaviours heightens the risk of developing numerous non-communicable diseases (NCDs), including diabetes, hypertension, cardiovascular disease, mental ill-health and overweight including obesity [15]. Moreover, there are increasing public health concerns about the concurrent rise in overweight/obesity and mental health ill-health in Sweden and globally [13, 14]. Overweight and obesity have also been independently linked to adverse mental health outcomes including depression and anxiety [21–23]. Although less researched, accumulating evidence suggest that diet and nutrition are significantly associated with mental health and well-being [16, 23–29]. Notably, unhealthy diets rich in processed and fried foods, consisting of sugary snacks, and SSBs are linked to an elevated risk of mental health ill-health, such as stress [30], depression and anxiety [27]. In contrast, a healthy diet, characterized by high intake of fruits, vegetables, whole grains, and lean proteins, have been associated with better mental health including lower levels of stress, anxiety, depression and higher lfe satisfaction [25, 27, 31].

Despite increasing research concerning the impact of dietary behaviours on health, there are significant knowledge gaps. It is well documented that the quantity and quality of foods consumed may have an impact on physical health including overweight/obesity, however, less is known about the association between meal patterns (such as the regularity of breakfast and family meals) and overweight/obesity among Swedish adolescents. Furthermore, although numerous studies have explored the relationship between various aspects of dietary behaviours and mental health and well-being among adults [18, 31], there exists a notable paucity of research focused on children and adolescents. There is good evidence on socioeconomic inequalities in dietary behaviours [12, 32]. Meanwhile, the evidence is less clear on how socioeconomic inequalities in dietary behaviours contribute to the patterning of diet-related health outcomes among adolescents.

To address these gaps in the literature, our study aims to investigate the association between dietary behaviours, overweight/obesity, and mental health and well-being (including psychosomatic complaints, life satisfaction, and school-related pressure) among ≈11, ≈13 and ≈ 15-year-old Swedish adolescents.

## Materials and methods

### Participants and study design

The analyses were based on data from the 2017/2018 Swedish component of Health Behaviour in School-aged Children (HBSC) study, collected by the Public Health Agency of Sweden in conjunction with Statistics Sweden (SCB). HBSC study collects information on a comprehensive range of factors — including health, education, social, and family aspects — that influence young people’s health and well-being. A nationally representative sample of 450 schools with grades 5, 7 and 9 were randomly selected. Then a single class from one grade (corresponding to ≈ 11, ≈13 and ≈ 15-year-olds) was randomly selected from each school. Approximately 4294 students from 213 schools responded to the questionnaire, for an individual participation rate of 89%. Due to incomplete information on some items, the final analytical sample consisted of 3692 adolescents (Appendix Fig. A1), of which there were 1,844 (49.9%) boys and 1,848 girls (50.1%).

### Measurements

#### Outcomes

**Weight status** was characterised according to the 2007 World Health Organization (WHO) growth reference standards for children and adolescents 5–19 years old [33]. Using standardized z-scores for body mass index (BMI) based on self-reported height and weight, overweight was coded as equivalent to BMI values at + 1 standard deviation (SD) and obesity equivalent to BMI values at or above + 2 SD value.

**Mental health and well-being** were assessed using three complementary measures that address distinct yet overlapping facets. ***Multiple psychosomatic health complaints*** was based on the frequency with which respondents experienced the following eight health complaints over the past 6 months: feeling low, irritability or bad temper, feeling nervous, difficulties in getting to sleep, feeling dizzy, headache, stomach ache, and backache, with the following response options: “about every day”, “more than once a week”, “about every week”, “about every month”, and “rarely or never”. In line with recommendations from the HBSC network, we dichotomised the measure to indicate those adolescents experiencing two or more of the eight symptoms at least once a week [33]. This measure has been adequately tested, and it has been shown to be a reliable and valid instrument for adolescent populations [34, 35]. The cronbach’s alpha was 0.84, indicating that the items have a high internal consistency. ***Life satisfaction*** was captured using Cantrils ladder, which lies on a scale from 0 to 10 (10 representing the best possible life) [36]. High life satisfaction was defined as a score of 6 or above. The measure has been shown to be a reliable and valid instrument to measure overall mental well-being among adolescents [37, 38]. ***School-related pressure*** was included in this study as a proxy for feelings of general stress. Students were asked “How pressured do you feel by the schoolwork you have to do?” with four response options: “not at all”, “a little”, “some” and “a lot”. Consistent with previous international reports and publications from the HBSC study [39, 40], these answers were categorized as pressured (answer options 3 and 4) versus not pressured (answer options 1 and 2). The measure is well functioning and has been used widely [39, 41].

#### Exposures

Three key measures of dietary behaviours were examined in this study.

1. **Meal patterns were assessed with two measures**, (i) *Daily breakfast consumption* during school days was measured with responses to the question: “How often do you eat breakfast (more than a glass of milk or juice) on weekdays?” The response options ranged from “5 weekdays” to ”never” having breakfast on weekdays”. This indicator was dichotomised into “daily” (5 days a week) and “not daily” (less than 5 days a week) breakfast consumption; and (ii) ***Daily family meals*** measured with responses to the question: “How often do you and your family usually have meals together? ”. The responses provided were “Every day”, “5–6 days a week”, “3–4 days a week”, “1–2 days a week” and “less than once a week”. This was then dichotomized into “daily” vs “not daily”.
2. **Diet quality was measured by the consumption frequency of four food items**, these were: (i) fruits; (ii) vegetables; (iii) sugar sweetened beverages (SSBs) and; (iv) sweets. Participants were asked: “How many times a week do you usually eat or drink …. ?” and provided with the following responses: “Never”, “Less than once a week”, “Once a week”, “2–4 days a week”, “5–6 days a week”, “Once a day/every day” and “More than once per day”. The consumption of fruit and vegetables was then dichotomised into “daily” vs. “not daily”. Previous literature coded SSBs and sweets as daily vs. not daily consumption [42, 43]. However, in this current study, these variables were dichotomized and coded as “more than once per week” vs “once per week or less” to reflect Swedish dietary traditions of saturday candy (lördagsgodis) and cosy Fridays (fredagsmys) [44]. All other measures were coded in line with (inter)national and HBSC reporting standards [42, 45].
3. **Healthy dietary behaviours**. Using the above 6 dietary indicators, a global score was created by counting the number of individual dietary behaviours. The meaure was based on the sum of the six dietary indicators (breakfast (yes = 1, no = 0), family meals daily (yes = 1, no = 0), fruit daily (yes = 1, no = 0), vegetable daily (yes = 1, no = 0), SSBs once per week or less (yes = 1, no = 0) and consumption of sweets once per week or less (yes = 1, no = 0). The responses ranged from 0 to 6. They were then dichotomized, with a value of one indicating three or more healthy dietary behaviours.

#### Confounders

The socioeconomic and demographic confounders selected for use in this study, were based on earlier literature suggesting associations with both dietary behaviours and health [12, 32, 46]. The coding of these measures reflect those of earlier studies and international standards [45, 47, 48]. The measures were *Migrant status*, categorised into “Both Swedish parents”, “One foreign parent”’ and “Both foreign parents”. *Family structure* categorised as residing with “Both parents”, “Single parent”, “Other family types”. The socioeconomic status of the respondents and their families was assessed using the Family Affluence Scale (FAS). This scale is based on the sum score of six questions measuring a family’s absolute wealth, these were:

1. Does your family own a car or another motorized vehicle? (No = 0; Yes, one = 1; Yes, two or more = 2).
2. Do you have your own bedroom? (No = 0; Yes = 1).
3. How many computers (including laptops and tablets, but not game consoles and smartphones) does your family own? (None = 0; One = 1; Two = 2; More than two = 3).
4. How many bathrooms (rooms with a bath/shower or both) are in your home? (None = 0; One = 1; Two = 2; More than two = 3).
5. Does your family have a dishwasher? (No = 0; Yes = 1).
6. How many times did you and your family travel out of Sweden for holiday/vacation last year? (Never = 0; Once = 1; Twice = 2; More than twice = 3).

The responses were categorised using the following scores: 0 − 7 (low FAS), 8 − 11 (medium FAS), and 12 − 13 (high FAS).

*Perceived family wealth*, which is a relative measure of family wealth was also included in the analyses. Respondents were provided with five categories, “Very well off”, “Quite well off”, “Average”, “Not so well off” and “Not at all well off”. This measure was dichotomized into “well off” (“very well off”, “quite well off”) vs “not well off” (“average”, “not so well off” and “not at all well off”). *Parental employment* was created using two survey questions which asked respondents about their mothers and fathers employment status respectively. The measure was categorised as “Both parents work”, one parent works”, “No parents work”. Finally, gender and age were included in the analyses.

## Statistical analyses

The analyses were carried out in four steps and were guided by the directed acyclic graph (DAG) in appendix Figure A2. The DAG shows the proposed associations between dietary behaviours (exposure), weight status and mental health and well-being (outcome) adjusting for the socioeconomic status and demographic characteristics of adolescents and their families (confounders). We first provided descriptive statistics of our exposure and outcome variables. We then proceeded to examine the prevalence of dietary behaviours across the socioeconomic, demographic variables and health outcomes. We further estimated, using logistic regression, the bivariate associations between dietary behaviours and socioeconomic and demographic factors. Lastly, separate multivariate logistic regression models were fitted to assess the association between (i) dietary behaviours and overweight/obesity; as well as (ii) mental health and well-being, with and without adjustment, for socioeconomic status and demographic factors. The results are presented as odds ratios (OR) with 95% confidence intervals (CI). Python version 3.10 was used for all statistical analyses. Missing data were excluded from the statistical analyses but were presented in the descriptive statistics.

## Results

### Descriptive statistics

#### Final study sample

Table 1 presents the socioeconomic, demographic, exposure and outcomes variables for the sample. Most participants reported medium (49.1%) to high family affluence (42.9%), aligning with 81.1% who perceived their family as ‘well off’. The majority lived with both parents (65.1%) and had both working parents (82.9%), and had two Swedish parents (64.8%). Regarding the outcome measures, 12.3% of the sample were overweight and 5.5% had obesity. While 82.2% reported high life satisfaction, 52% reported having two or more psychosomatic health complaints more than once per week, whereas 48.1% reported having two or less. Regarding school-related pressure, 57.7% of students indicated they felt no pressure related to school, whereas 39.9% reported feeling pressured. Missing data spanned from 1.5% to 10.8% across all the variables included in the study.

**Table 1:** Sociodemographic and health characteristics of the study sample

	Freq.	%
**Gender**		
Boys	1844	49,9
Girls	1848	50,1
**Age**		
11	1001	27,1
13	1245	33,7
15	1446	39,2
**Family Affluence Scale (FAS)**		
Low FAS	191	5,2
Medium FAS	1811	49,1
High FAS	1584	42,9
Missing	106	2,9
**Perceived family wealth**		
Well off	2994	81,1
Not well off	623	16,9
Missing	75	2,0
**Family situation**		
Both parents	2403	65,1
Single-parent	268	16,8
Other	622	7,3
Missing	399	10,8
**Parental employment**		
Both parents work	3061	82,9
one parent works	328	8,9
No parents work	55	1,5
Missing	248	6,7
**Migrant status**		
Both parents Swedish	2391	64,8
One foreign parent	737	20,0
Both parents foreign	488	13,2
Missing	76	2,1
**Weight status (BMI)**		
Overweight	455	12,3
Obesity	204	5,5
Underweight	123	3,3
Normal Weight	2554	69,2
Missing	356	9,6
**Life satisfaction**		
Low (0–5)	513	13,9
High (6-10)	3 036	82,2
Missing	143	3,9
**Psychosomatic complaints**		
≥ 2 complaints	1 917	52,0
≤ 1 complaints	1 775	48,0
**School-related pressure**		
Not pressured	2130	57,7
Pressured	1473	39,9
Missing	89	2,4

#### Prevalence of dietary behaviours by socioeconomic status and demographic factors

**Fig. 1:**
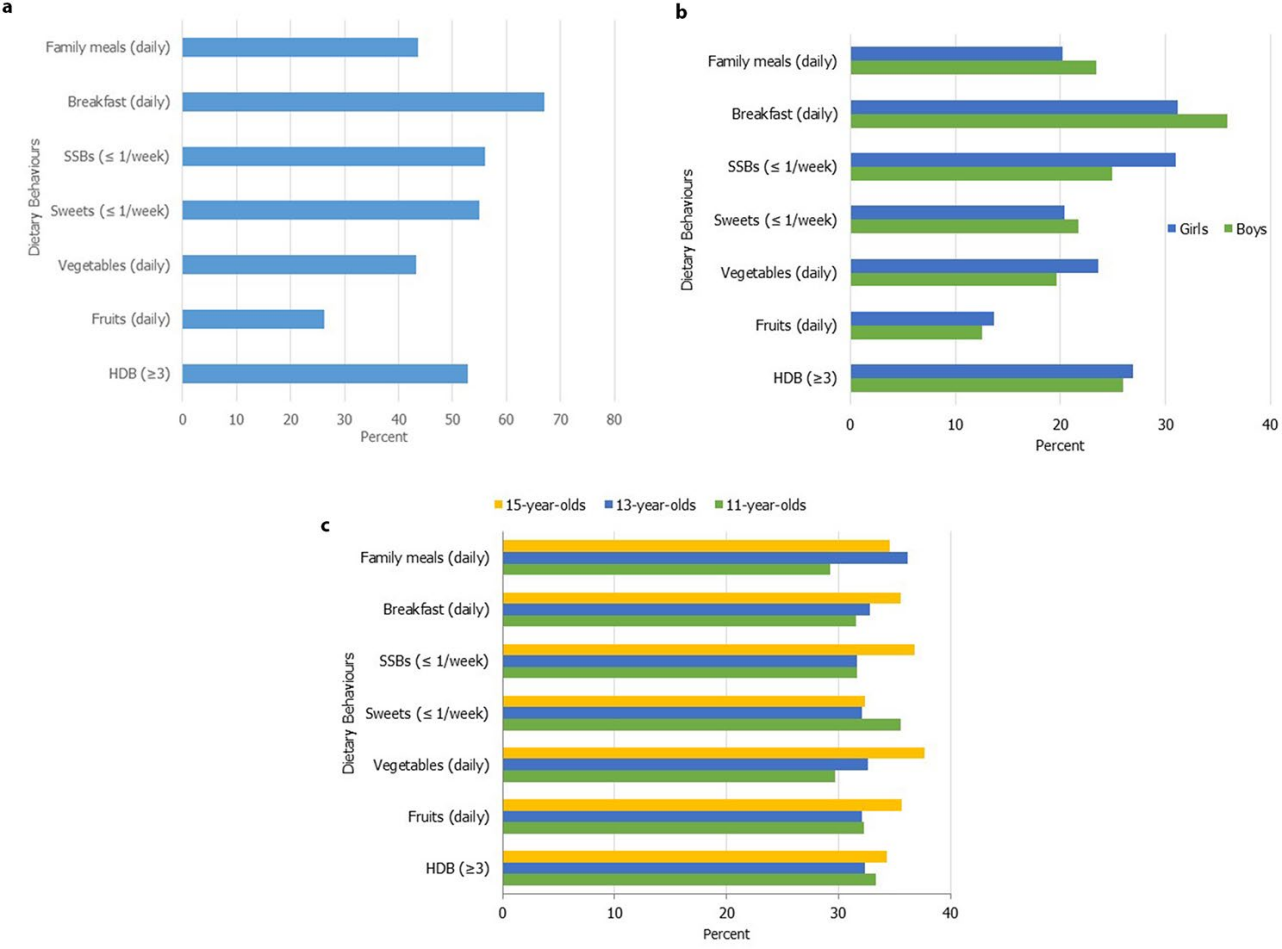
(**a)** Prevalence of dietary behaviours for the total sample. (**b**) Distribution of dietary behaviours by gender. (**c**) Distribution of dietary behaviours by age. *Notes*: The figures b and c reflect the distribution of the adolescents who engaged in a particular dietary behaviour. Sugar Sweetened Beverages (SSBs), Healthy Dietary Behaviours (HDBs)

We examined the prevalence of dietary behaviours across socioeconomic status and the demographic factors included in the study. Figure 1a, b and c present the prevalence of dietary behaviours for the total sample, and the distribution by gender and age. The results indicate that  53% of adolescents followed the recommendations for at least three dietary behaviours, of these 26% were boys and 27% were girls (figure 1b). The results further indicated that the prevalence in the consumption of family meals, breakfast and sweets once per week or less were higher among boys. In contrast, girls had higher prevalence rates in the consumption of fruits, vegetables and SSBs once per week or less. Dietary behaviours appear to remain relatively stable across age for both boys and girls (fig. 1c).

There was a socioeconomic gradient in the dietary patterns of adolescents. Adolescents from two parent households, those with two Swedish parents, who had both parents employed, and those perceiving their families as “well off” often engaged in more healthy dietary behaviours, with a higher consumption of fruits and vegetables, breakfast and family meals. Also, these groups engaged in some unhealthy dietary practices with higher consumption rates of sweets and SSBs, maximum once per week. Indicating therefore that children from lower socioeconomic groups consumed higher rates of sweets and SSBs. A key exception was among adolescents classified as having high FAS, they had a lower prevalence of consuming sweets and SSBs, compared to with adolescents with medium and low FAS scores. The full results can be found in Table SA1.

### Multivariate Logistic regression

#### Dietary behaviours, socioeconomic and demographic confounders

**Fig. 2:**
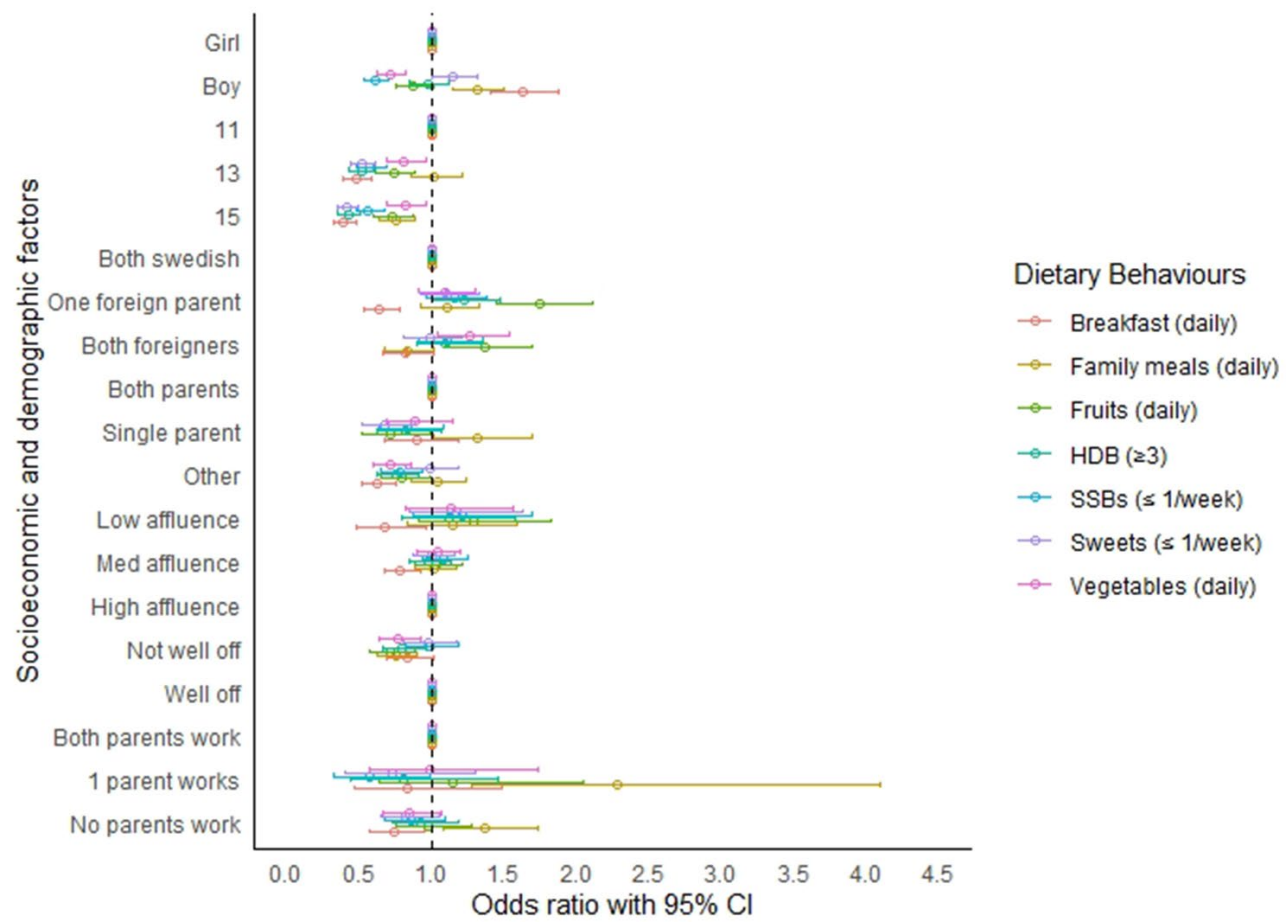
Association between dietary behaviours and socioeconomic status and demographic characteristics of adolescents 11-, 13- and 15-years-old. *Notes*: The results are based on the odds ratios from logistic models. Meal patterns (eating breakfast and family meals) and the consumption of fruits and vegetables were measured as daily intake; consumption of sugar sweetened beverages (SSBs) and sweets were assessed as once per week or less

In Fig. 2, we present the results of the bivariate logistic regression models. We found that boys were more likely than girls to consume daily breakfast (OR = 1.62, CI: 1.40–1.88) and family meals (OR = 1.31, CI: 1.15–1.50) but they consumed fruits (OR = 0.87, CI: 0.75–1.01) and vegetables (OR = 0.71, CI: 0.62– 0.81) less frequently. Older children, particularly 15-year-olds, had lower daily breakfast intake, and they were less likely to eat family meals (OR = 0.75, CI: 0.63-088). Socioeconomic status such as low (OR = 0.68, CI: 0.48–0.96) and medium (OR = 0.78, CI: 0.67–0.92) FAS scores and the perception that ones family was ”not well off” (OR = 0.84, CI: 0.69–1.01), were associated with irregular breakfast but not statistically significant with the other dietary behaviours. Similarly, adolescents with unemployed parents (OR = 0.74, CI: 0.57– 0.95) and those from single-parent households (OR = 0.89,CI:0.67–1.19) were less likely to consume daily breakfast. Dietary behaviours also varied by migrant status. Adolescents with one parent (OR = 1.74, CI: 1.44–2.11) or both parents (OR = 1.36, CI: 1.09–1.70) with a foreign background consumed more fruits but missed breakfast more frequently compared to adolescents with two Swedish parents. Full results, Table SA2.

#### Health and dietary behaviours

Table 2 shows the association between dietary behaviours, overweight/obesity and mental health and well-being. Regular family meals, daily fruit and vegetable intake were consistently associated with positive health outcomes. Specifically, daily family meals were associated with higher life satisfaction (OR = 1.55, CI: 1.27–1.88), fewer psychosomatic complaints (OR = 0.78, CI: 0.69–0.89), and lower school-related pressure (OR = 0.67, CI: 0.58–0.77). Starting the day with breakfast had notable benefits. It was associated with higher life satisfaction (OR = 2.34, CI: 1.90–2.88), reduced psychosomatic complaints (OR = 0.45, CI: 0.39–0.52), and lower perceived school-related pressure (OR = 0.57, CI: 0.49–0.67). Similarly, consumption of sweets and SSBs once per week or less was associated with higher life satisfaction (SSBs OR = 1.27,CI:1.04–1.55)], and a lower likelihood of experiencing two or more psychosomatic health complaints in a week [(SSBs OR:0.68,CI:0.60–0.79); sweets OR = 0.74,CI:0.64–0.85)] and school-related pressure (SSBs OR: 0.83;CI:0.72–0.97). When accounting for the confounders, the impact of fruits and vegetables on mitigating school-related pressure is not evident, and the association between consuming sweets and both school-related pressure and life satisfaction is attenuated.

An assessment of the results related to weight status, indicated that daily breakfast (OR = 0.71 CI: 0.57–0.88) and vegetable (OR = 0.71 CI: 0.58–0.88) intake was associated with a lower likelihood that adolescents would be living with overweight. A lower likelihood of obesity was also found among adolescents who consumed breakfast (OR = 0.56 CI: 0.41–0.76) on a daily basis but was significantly higher with consumption of sweets (OR = 1.30 CI: 1.14–2.05) at least once per week. However, the other dietary behaviours were not significantly associated with overweight and obesity.

**Table 2:** Association between dietary behaviours, overwight/obesity and mental health and well-being among adolescents 11, 13 and 15-years-old

		Unadjusted models			Adjusted Models		
		Odds ratio	95% CI		Odds ratio	95% CI	
**High Life satisfaction**							
Breakfast (daily)		2.88	2.37	3.49	2.31	1.88	2.84
Family meals (daily)		1.55	1.27	1.88	1.41	1.14	1.73
Fruits (daily)		1.53	1.21	1.93	1.42	1.12	1.82
Vegetables (daily)		1.55	1.28	1.89	1.48	1.21	1.82
SSBs (≤ 1/week)		1.27	1.05	1.53	1.27	1.04	1.55
Sweets (≤ 1/week)		1.10	0.91	1.34	0.96	0.78	1.18
HDB (**≥** 3)		1.94	1.60	2.36	1.68	1.36	2.07
**Psychosomatic health complaints (** **≥** ** 2)**							
Breakfast (daily)		0.41	0.35	0.47	0.45	0.38	0.52
Family meals (daily)		0.78	0.69	0.89	0.84	0.74	0.97
Fruits (daily)		0.82	0.71	0.95	0.84	0.72	0.98
Vegetables		0.79	0.69	0.90	0.77	0.68	0.89
SSBs (≤ 1/week)		0.74	0.65	0.85	0.68	0.60	0.79
Sweets (≤ 1/week)		0.70	0.61	0.80	0.74	0.64	0.85
HDB (**≥** 3)		0.60	0.52	0.68	0.62	0.54	0.72
**School-related pressure**							
Breakfast (daily)		0.45	0.39	0.52	0.57	0.49	0.67
Family meals(daily)		0.67	0.58	0.77	0.76	0.66	0.89
Fruits (daily)		0.84	0.72	0.97	0.91	0.77	1.09
Vegetables (daily)		0.89	0.78	1.02	0.88	0.76	1.03
SSBs (≤ 1/week)		0.82	0.72	0.94	0.83	0.72	0.97
Sweets (≤ 1/week)		0.67	0.58	0.77	0.86	0.74	1.00
HDB (**≥** 3)		0.58	0.50	0.67	0.70	0.60	0.82
**Overweight**							
Breakfast (daily)		0.74	0.60	0.91	0.71	0.57	0.88
Family meals (daily)		0.96	0.79	1.18	0.92	0.75	1.13
Fruits (daily)		0.86	0.68	1.08	0.82	0.64	1.03
Vegetables (daily)		0.70	0.57	0.86	0.70	0.57	0.87
SSBs (≤ 1/week)		0.92	0.75	1.12	0.93	0.75	1.14
Sweets (≤ 1/week)		1.21	0.99	1.48	1.16	0.94	1.42
HDB (**≥** 3)		0.86	0.70	1.06	0.83	0.68	1.03
**Obesity**							
Breakfast (daily)		0.65	0.49	0.88	0.56	0.41	0.77
Family meals (daily)		1.28	0.96	1.71	1.22	0.91	1.63
Fruits (daily)		1.13	0.82	1.54	1.17	0.85	1.61
Vegetables (daily)		0.95	0.72	1.27	0.99	0.74	1.33
SSBs (≤ 1/week)		1.21	0.90	1.63	1.29	0.95	1.75
Sweets (≤ 1/week)		1.63	1.23	2.18	1.52	1.13	2.04
HDB (**≥** 3)		1.07	0.80	1.44	1.02	0.75	1.39

*Notes*: 1. The confounders included in the adjusted models were sex, age, migrant status, family structure, FAS, perceived family wealth and parental employment. Sugar Sweetened Beverages (SSBs), Healthy Dietary Behaviours (HDB)

## Discussion

This study contributes to the relatively understudied literature assessing the association between dietary behaviours, overweight/obesity, as well as mental health and well-being among adolescents. To our knowledge, this study is the first to analyse this relationship using the Swedish HBSC dataset. One additional novel feature of our work is that we have shown that meal patterns, that is the regularity with which adolescents consume breakfast and family meals, may be as important as diet quality measured as the frequency with which fruits, vegetables, sweets and SSBs are consumed. Furthermore, this is one of the first Swedish studies to explore the relationship between meal patterns and overweight/obesity among a nationally representative sample of adolescents 11–15 years old.

In relation to our key study aims, we observed that healthier dietary behaviours, such as the daily consumption of fruits, vegetables, family meals and breakfast, were associated with better mental health and well-being. Specifically, we found that adolescents who engaged in healthy behaviours were more likely to be satisfied with their lives, had lower psychosomatic complaints and school-related pressure. These relationships remained significant across the various health measures assessed, even after adjustment for potential confounders. We consistently observed that daily consumption of breakfast and family meals is linked to a higher likelihood of better mental health and well-being. However, the relationship between mental health and well-being and the consumption of fruits and vegetables showed some variability. These results were similar to those of previous studies assessing the relationship between dietary behaviours and mental health and well-being [16–18].

Although the results related to the consumption of SSBs and sweets were less consistent, the overall results indicated that consuming SSBs once per week or less was associated with higher life satisfaction, while the consumption of sweets and SSBs were both associated with a lower likelihood for adolescents to report two or more psychosomatic complaints more than once in a week and school-related pressure. These findings are similar to previous studies suggesting that sweets and SSBs was associated with poor health [30]. However, adolescents may potentially use these types of foods as a coping strategy for dealing with symptoms of mental ill-health, such as anxiety and stress [49–51].

Our findings further suggest that daily breakfast consumption is associated with a lower likelihood of overweight or obesity among adolescent. This finding echoes that of earlier studies indicating that breakfast may be a protective factor for unhealthy weight status and other negative health outcomes [6, 21, 26]. However, other dietary behaviours such as the consumption of fruits, SSBs and regular family meals did not show any significant associations with overweight or obesity. These findings are a departure from previous studies indicating that family meals [19] reduces the risk of obesity, and engaging in unhealthy dietary behaviours – low in the consumption of fruits and vegetables but high in the consumption of SSBs and sweets – is associated with overweight and obesity [13, 52]. It is worth noting that maintaining a healthy weight is a complex issue influenced through a complex interaction of genetic, environmental, psychosocial and behavioural factors [52].

The results of our study may have important implications for overweight/obesity, as well as mental health and well-being of adolescents. Similar to previous studies [11, 12], we found that the dietary behaviours of Swedish adolescents do not align with national recommendations [7]. Our findings also suggest that addressing the socioeconomic and demographic factors contributing to inequalities in health outcomes associated with dietary behaviours may reduce the risk of overweight/obesity while simultaneously increasing the likelihood of good mental health and well-being. This is because, consistent with previous studies [14, 15, 17, 29, 43, 47] we found that dietary behaviours vary across populations based on age, gender and socioeconomic status. Notably, boys tended to consume breakfast and partake in family meals more frequently than girls but consumed fruits and vegetables less frequently. Furthermore, adolescents from families with higher socioeconomic status, those with specific demographic attributes such as two-parent households and two Swedish-born parents, were more likely to engage in favourable dietary behaviours. These results echo those of earlier studies [17, 29, 47].

One should also keep in mind that a more holistic view of dietary behaviours may need to be adopted, given that previous studies have shown that there are significant associations between meal patterns and diet quality [10, 12, 13]. In addition, regular consumption of family meals and breakfast, may be indicators of well-functioning families, and that this may also influence mental health and well-being, as well as actual nutritional intake [20]. Moreover, at least one previous study showed that more frequent family dinners were associated with better mental health among adolescents. The association was partly attributable to improved parent-adolescent communication. Additionally, the health benefits appeared to be consistent across age groups and levels of family affluence [20]. The developmental stage of the study population may explain some of the observed findings. Prior research suggested that changes in dietary behaviours can occur naturally with age [43, 53]. As adolescents grow older, breakfast consumption might decline due to competition with their sleeping time, while increasing autonomy from parents is a typical developmental milestone, and this could contribute to fewer family meals [43].

Overall, the results from our study suggest that adopting healthy dietary behaviours have the potential to mitigate some of the risks related to overweight, obesity, and to improve the mental health and well-being of adolescents. Moreover, the findings underscore the significance of gaining a deeper insight into methods of improving mental health and well-being, as well as addressing the escalating rates of mental illness and issues with overweight and obesity in adolescents – which are key public health priorities [54]. Implementing policies that promote the intake of fruits and vegetables, decrease the consumption of SSBs and sweets, and emphasize the value of having breakfast and sharing family meals could offer a cost-effective public health intervention.

### Strengths and limitations

We have identified several strengths and limitations that should be considered when interpreting the findings and evaluating the results of this study. One key strength is the utilization of a large, representative population-based sample of Swedish adolescents, allowing for direct comparisons across various socioeconomic and demographic groups. The use of random sampling in the data collection process [45] enhances the statistical power and generalizability of the findings. Moreover, the selected survey questions have been derived from previous national studies and the HBSC international collaboration. The questions have therefore been tested across different populations, and have shown to be valid and reliable measures [45].

Another strength is the inclusion of socioeconomic and demographic confounders such as sex, age, parents’ country of birth, family type, FAS, perceived family wealth, and parents’ employment status in the regression models, which helps to minimize confounding effects and strengthens the validity of the associations examined.

Several limitations must be acknowledged. There are inherent limitations stemming from the use of cross-sectional data. This curtails our ability to deduce causal relationships and delve into complex associations (such as reverse causality, bi-directionality, and endogeneity) which are potentially present among the studied measures. For instance, the observed associations may have arisen from a relationship where the mental health and well-being of the adolescents determines their dietary behaviours. For example, as discussed above, it has been shown that stress is a key driver for unhealthy dietary behaviours, increasing the risk of consuming utraprocessed foods - sugary snacks, SSBs, packaged baked goods and ready meals - which are high in calories, sugar and fat [49–51]. Another limitation associated with using cross-sectional data is the issue of endogeneity. This arises due to the fact that respondents report their health and diet concurrently, this may contribute to potential correlations between these two responses. To comprehensively tackle these limitations, future research may benefit from using longitudinal data and/or intervention studies.

Furthermore, the HBSC study does not probe deeply into granular nutritional intake details. It focuses on the frequency of consumption for fruits, vegetables, SSBs, and sweets, without quantifying specific amounts, portion sizes, or calorie intake. Despite this constraint, the questionnaire is widely utilised and has been validated for assessing adolescents’ dietary patterns [14, 32, 42, 43, 45]. It is also crucial to highlight that these measures serve as indicators of healthier dietary behaviours and overall diet quality. Moreover, studies have shown that health behaviours tend to be closely interrelated [48]. For instance, the consumption of breakfast and family meals is often significantly linked to increased intake of fruits and vegetables [5].

Further research is necessary to provide clarity on specific study outcomes. In particular, future investigations should delve deeper into the complex relationships between dietary behaviours, weight status, and mental health. Understanding these complex interrelated issues is vital for creating comprehensive strategies to address the converging health issues faced by adolescents. This is because numerous factors shape dietary behaviours, including family and peer consumption patterns, food availability, broader food environments, and exposure to advertisements and marketing [1–3]. It is essential for future studies to disentangle the socioeconomic determinants that influence dietary behaviours and to understand the health disparities connected to these factors. Knowledge on these issues is essential for developing effective and targeted interventions, especially for individuals from low-income and vulnerable groups.

## Conclusion

Dietary behaviours are associated with weight status, and the mental health and well-being of adolescents; and these findings are largely consistent with existing research highlighting the importance of a balanced and nutritious diet. Our examination of the associations between dietary behaviours and adolescent mental health and well-being adds new insights to an under-researched area of the literature. Furthermore, our results echo numerous studies highlighting the link between health behaviours and social determinants of health. This underscores the urgency of promoting healthy dietary behaviours, particularly among adolescents from vulnerable socioeconomic and demographic groups.

### Electronic supplementary material

Below is the link to the electronic supplementary material.

Supplementary Material 1

## Data Availability

Application and enquiries for data use should be sent to skolbarns.halsovanor@folkhalsomyndigheten.se.
