# Supplementary material for: Associations between dietary behaviours and the mental and physical well-being of Swedish adolescents
Source: Child Adolesc Psychiatry Ment Health. 2024 Mar 30;18:43. doi: 10.1186/s13034-024-00733-z (PMC10981827; doi:10.1186/s13034-024-00733-z)
Supplement: Supplementary file 1 — Supplementary Material 1 [file 13034_2024_733_MOESM1_ESM.pdf]

Supplementary Appendix: Associations between Dietary behaviors and Mental and Physical Health Among Swedish Adolescents: The Role of Socioeconomic and Demographic Status

Kenisha Russell Jonsson[1]. Cameron K Bailey. Maria Corell[1]. Petra Löfstedt[1]. Nicholas Kofi Adjei[234]

[1] School of Public Health and Community Medicine. Institute of Medicine. Gothenburg University. Box 463. 405 30. Göteborg. Sweden

[2] University of Liverpool. Department of Public Health and Policy. Waterhouse Building 2nd Floor . Block F. Liverpool . L69 3GL. UK

[3] Leibniz Institute for Prevention Research and Epidemiology - BIPS. Bremen. Germany

[4] Health Sciences Bremen. University of Bremen. Bremen. Germany

Corresponding author: Nicholas Kofi Adjei. University of Bremen. Bibliothekstrasse 1. 28359 Bremen. Email: *adjei@uni-bremen.de*

Figure A1. Data inclusion flow chart

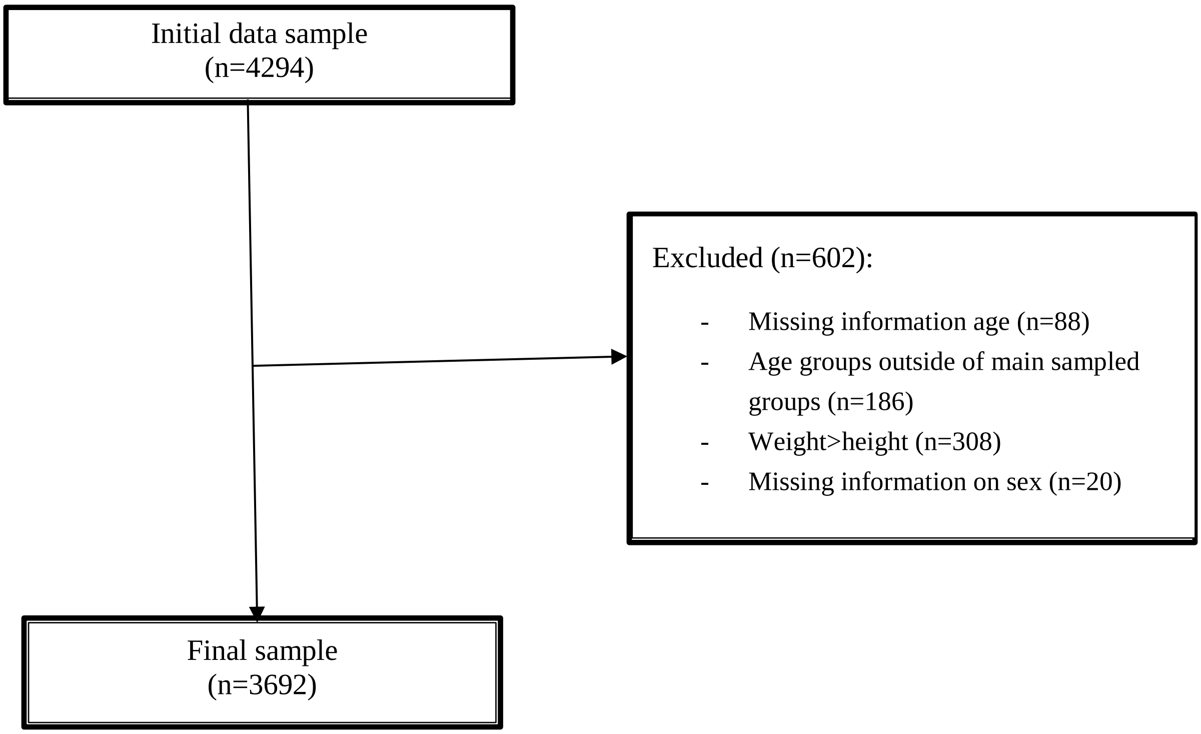

Figure A2. Directed acyclic graph (DAG) for the current study

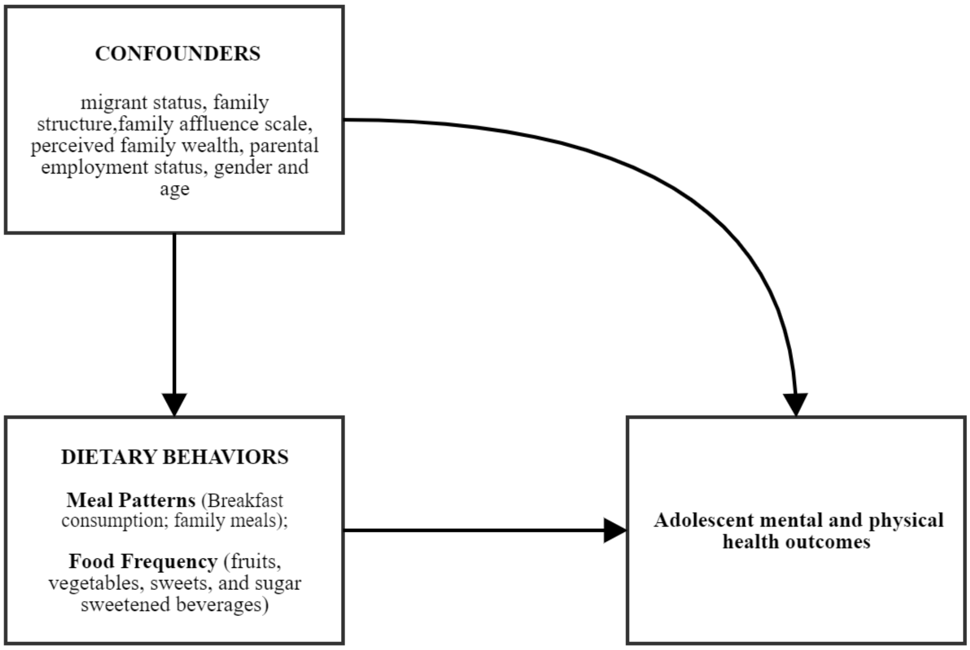

Table SA1. The Association between socioeconomic. demographic characteristics and dietary behaviours

	Breakfast			Family meals			Fruit			Vegetable			SSB			Sweets			Healthy dietary habits		
	Odds ratio	95% CI		Odds ratio	95% CI		Odds ratio	95% CI		Odds ratio	95% CI		Odds ratio	95% CI		Odds ratio	95% CI		Odds ratio	95% CI	
**Sex**																					
Girl	1			1			1			1			1			1			1		
Boy	1.62	1.40	1.88	1.31	1.15	1.50	0.87	0.75	1.01	0.71	0.62	0.81	0.61	0.53	0.70	1.15	1.00	1.31	0.97	0.85	1.12
**Age**																					
11	1			1			1			1			1			1			1		
13	0.48	0.39	0.59	1.02	0.86	1.21	0.73	0.61	0.89	0.81	0.68	0.96	0.57	0.48	0.68	0.52	0.43	0.61	0.51	0.43	0.62
15	0.39	0	0.47	0.75	0.63	0.88	0.72	0.60	0.87	0.82	0.69	0.96	0.56	0.47	0.67	0.41	0.35	0.49	0.42	0.35	0.50
**Migrant status**																					
Both Swedish	1			1			1			1			1			1			1		
One foreign parent	0.64	0.53	0.77	1.10	0.92	1.32	1.74	1.44	2.11	1.09	0.91	1.30	1.15	0.96	1.38	1.11	0.92	1.33	1.22	1.01	1.47
Both foreigners	0.82	0.66	1.02	0.83	0.68	1.02	1.36	1.09	1.70	1.26	1.04	1.54	1.11	0.91	1.35	0.99	0.81	1.21	1.09	0.89	1.35
**Family type**																					
Both parents	1			1			1			1			1			1			1		
Single parent	0.89	0.67	1.19	1.31	1.02	1.70	0.72	0.52	0.99	0.88	0.68	1.14	0.83	0.64	1.07	0.68	0.52	0.89	0.82	0.63	1.07
Other	0.62	1	0.75	1.03	0.86	1.24	0.80	0.65	0.98	0.71	0.59	0.86	0.78	0.65	0.94	0.98	0.82	1.18	0.75	0.62	0.91
**FAS**																					
High affluence	1			1			1			1			1			1			1		
Med affluence	0.78	0.67	0.92	1.01	0.88	1.17	1.03	0.88	1.21	1.04	0.90	1.19	1.08	0.94	1.24	1.00	0.87	1.16	0.97	0.84	1.13
Low affluence	0.68	0.48	0.96	1.15	0.83	1.59	1.29	0.91	1.82	1.14	0.82	1.57	1.21	0.87	1.69	1.17	0.84	1.62	1.12	0.79	1.58
**Perceived family wealth**																					
Well off	1			1			1			1			1			1			1		
Not well off	0.84	0.69	1.01	0.75	0.62	0.90	0.71	0.57	0.88	0.77	0.64	0.92	0.99	0.82	1.18	0.98	0.81	1.18	0.80	0.66	0.96
**Parental employment**																					
Both parents work	1			1			1			1			1			1			1		

1 parent works	0.83	0	1.49	2.28	1.27	4.09	1.14	0.64	2.04	0.99	0.56	1.74	0.57	0.32	0.99	0.72	0.40	1.30	0.80	0.45	1.45
No parents work	0.74	0.57	0.95	1.36	1.08	1.73	0.98	0.75	1.27	0.84	0.66	1.07	0.85	0.67	1.09	0.82	0.64	1.05	0.93	0.72	1.19
